# Should the Probationary System Be Revolutionised?

**Published:** 1919-07-12

**Authors:** 


### THE MAGIC OF EDUCATION.

### Should the Probationary System be Revolutionised?

MAJOR HUNT showed his shrewd perception when he observed in Parliament that "there is no magic in Registration." He might have added that there *is* magic in education, a fact which Miss Cox-Davies fully appreciated. I have been wondering what Miss Cox-Davies had in mind when she expressed something more than a hope that the future would witness the establishment of a Chair of Nursing in one of the women's colleges. Doubtless this eminently practical lady had in view something precise and definite, although not necessarily worked out in detail.

### UNIVERSITY DIPLOMAS IN NURSING.

Women's colleges, and likewise men's, where there are "chairs" of learning, are, as everybody knows, usually University Colleges, and lectures delivered there are intended for persons seeking or holding University degrees. In certain specific subjects, in which degrees are unobtainable, such as public health and teaching, diplomas are available, but these are awarded only to graduates. I take it, therefore, that Miss Cox-Davies looks forward to seeing University Diplomas in Nursing awarded to women holding degrees in arts or science. What is not yet clear, however, is whether it is contemplated that such distinctions shall be possible for the rank and file, or for instructors of the rank and file, such persons, for example, as might win College of Nursing Scholarships. In either case the idea is excellent, and is bound to produce the highest results.

### IS NURSING A PROFESSION?

It may be in the memory of HOSPITAL readers that some time ago I raised the question whether or not nursing is, properly so called, a profession, or whether it is, in strict accuracy, only an occupation. The desire and ambition of every nurse is, of course, that nursing should be, beyond dispute, a profession wherein women of talent and refinement might find scope for their abilities. The University is not a *sine qua non*. A man, and in some cases a woman, may become a solicitor, a barrister, an engineer, a veterinary surgeon, a chartered accountant, a medical practitioner, a teacher, a chemist—and yet have no University connection whatsoever. But in every instance a certain educational standard must be reached at entry, and in every instance there must be a period of training followed by a final qualifying examination. These conditions do not obtain in nursing. The probationer is not a pupil in the full sense. She is earning her living, and her services are of financial value to the hospital to which she is attached. Such accomplishments as English, arithmetic, not to mention the rudiments of Latin, French, and mathematics, do not come into consideration, and, if they did, with the outlook which nurses have so far enjoyed, there would be an almost complete absence of recruits. The question, fundamentally, is one of economics. It is perfectly true that many trained nurses possess high educational attainments. The Secretary to the Dublin Branch of the College of Nursing is a trained nurse, and also a distinguished University graduate. But these are exceptions, and I regret to observe that not only in this country, but in America, there are doctors, even in this enlightened age, who openly confess that they do not favour a high standard of education for nurses. It is difficult to understand the mentality of such men. They ought to be in dodo-land, but the fact remains.

### THERE IS MAGIC IN EDUCATION.

In the articles which I wrote, and to which I now refer, my plea was for a revolution of the probationary system. There is often a great deal wrapped up in a word, and I do not like the word "probationer." I prefer the word "nurse-pupil," which indicates a person in training. A "training-school" for a "probationer" is to some extent a contradiction of terms. I am trying to get down to bed-rock. A young woman enters a Metropolitan hospital as a probationer, and she has to work for anything between ten and twelve hours per diem. She is told that she is learning her profession at the expense of the hospital, and that she ought to be thankful if she receives small mercies. This is not quite true. She is learning, no doubt, but she is also working, and in a short time she becomes a cheap and efficient member of the staff. In fact, without such as she many hospitals would have to close their doors. In discussing this aspect of probation I seemed to be talking in the clouds. I felt that I was discussing an idea altogether Utopian and impossible. Now Miss Cox-Davies mentions a Chair of Nursing, and so I begin again to wonder, and likewise to hope. It is in the air as a post-war reform to bring the University to the poorest members of the community. The public are beginning to realise that there is magic in education. I am convinced, however, that the nursing world as a whole must remain outside such benefit unless the probationary system is revolutionised. If the nurse-probationer becomes a nurse-pupil she will be admitted at the age of seventeen or eighteen. She will require to present herself for an entrance examination in order to demonstrate that she possesses a sound elementary education. Her youth and energy will not be exploited in charity, but spent in undergoing a practical apprenticeship and in acquiring a higher education. Her salary may be nil, or only nominal, and at the age of twenty-one she will be a member of an honourable profession with a bright career in prospect. The present stupid hiatus between leaving school and beginning the work of life will disappear.

### BETTERMENT MUST BEGIN AT THE BOTTOM.

The majority of nurses, I am afraid, fail to realise that if the status of the profession is to rise, betterment must begin at the lowest rung of the ladder. If it ever happens that the nurse-probationer is superseded by the nurse-pupil, every trained nurse in the kingdom must automatically derive personal benefit from the change.

IERNE.

